# Local citrus sudden death-associated virus infection in Nicotiana benthamiana induces chloroplast structural changes and HR-like responses that do not restrict systemic virus movement

**DOI:** 10.1099/jgv.0.002252

**Published:** 2026-03-30

**Authors:** Sayuri Iwasaki, Dennis van Oevelen, Alba Pérez-Sánchez, Nell Lakatsch, Robin van der Helm, Lena Michailidou, Nadya N. Icksan, Bradley Shibata, Emilyn E. Matsumura

**Affiliations:** 1 Laboratory of Virology, Wageningen University and Research, 6700 AA 8 Wageningen, Netherlands; 2 Department of Cell Biology and Human Anatomy, School of Medicine, University of California, Davis, CA, USA

**Keywords:** citrus sudden death-associated virus, *Nicotiana benthamiana*, plant defence responses, plant viral infectious clone, virus–plant interactions

## Abstract

The use of infectious clones to establish virus infections in well-established model plants has allowed us to greatly advance our understanding of virus–host interactions, providing controlled systems to study viruses that naturally infect complex hosts such as fruit trees. Citrus sudden death-associated virus (CSDaV) is a *Marafivirus* associated with citrus sudden death disease in Brazil, yet its molecular interactions in citrus or model plant hosts remain poorly characterized. In this work, we investigated the interaction between CSDaV and the model plant *Nicotiana benthamiana*. We show that CSDaV establishes a symptomless systemic infection in *N. benthamiana* plants, while inducing a hypersensitive response (HR)-like cell death preceded by chloroplast abnormalities in locally inoculated leaves. Immunogold labelling showed that CSDaV virions were localized near or inside altered chloroplasts in locally inoculated leaves. Membranous vesicles at the periphery of chloroplasts were also observed, suggesting chloroplast-associated replication. However, trans-complementation assays in which the viral RNA-dependent RNA polymerase rescued replication of a non-replicative CSDaV did not confirm a clear association between the replication complex and chloroplasts. Expression analyses showed that while *HIN1*, an HR-associated gene, was upregulated in CSDaV locally infected leaves, reactive oxygen species (ROS) levels and *CAT1*, a gene encoding a ROS-scavenging enzyme, were significantly reduced in the same CSDaV-infected tissues compared to controls, suggesting an ROS-independent HR-like reaction. Furthermore, the salicylic acid (SA) pathway genes *PR1* and *ICS1* were not induced, suggesting suppression of SA-mediated systemic resistance. These findings demonstrate *N. benthamiana* as a systemic host plant for CSDaV and suggest an infection strategy that couples localized HR-like responses with systemic defence suppression.

## Introduction

The availability of viral infectious clones that can be used to infect experimental model plants such as *Nicotiana benthamiana* has significantly advanced our understanding of viral disease aetiology, virus biology and virus–host plant interactions in the past years [1]. Virus–host model systems have been instrumental in dissecting the molecular basis of virus–plant interactions and are particularly valuable for studying viruses that cause disease in complex plant hosts, such as perennial fruit trees like citrus. However, these systems have limitations, as viral infections are often initiated in non-natural ways (for example, by expressing viral RNA from a plasmid delivered via *Agrobacterium*) and typically involve non-natural plant hosts which are highly susceptible to viral infections. Such conditions do not always accurately reflect the natural virus–host relationship, especially when studying viruses that affect crops in agricultural contexts [1,2]. Nevertheless, understanding how virus–plant interactions develop in these model systems remains important, as they provide controlled environments to uncover conserved mechanisms and generate hypotheses that can later be tested in natural hosts and contexts.

Citrus-infecting viruses are examples of viruses from complex hosts whose study has greatly benefited from the development of infectious clones [3,8]. However, only a few of these have been shown to be infectious to experimentally used model plant hosts such as *N. benthamiana* [3,5]. Studies using an infectious clone of citrus tristeza virus (CTV; *Closterovirus tristezae*) in *N. benthamiana* plants, for example, have shown that CTV does not behave in the same way as it does in its natural citrus host. While CTV infection in citrus is restricted to the phloem, in *N. benthamiana* plants, it can also invade other tissues that include parenchyma and mesophyll cells [3]. Nevertheless, studies using this virus–host model system have enabled the unravelling of viral protein functions [9,10], mechanisms of virus-mediated RNA silencing suppression [11] and the intra-host genetic variability of CTV [12].

On the other hand, studies using an infectious clone of citrus sudden death (CSD)-associated virus (CSDaV; *Marafivirus citri*, family *Tymoviridae*) in *N. benthamiana* plants are limited. CSDaV was originally identified in citrus plants grown in the southwestern region of São Paulo state in Brazil [13]. Its presence has been strongly associated with CSD disease, which caused the death of approximately four million sweet orange (*Citrus sinensis* L. Osb.) trees in that region in the early 2000s [13]. However, more than 20 years after CSDaV was first reported, it remains unclear whether this virus is the definitive and the only causal agent of CSD. Its low titre and frequent co-infection with CTV in citrus plants, in addition to its unknown biological vector, have hindered efforts to establish a direct role in disease development. Furthermore, CSD symptoms typically take 2 years to develop [13,14], further complicating and prolonging studies on this pathosystem.

In previous work by the senior author, a CSDaV infectious clone was developed to overcome these challenges and to study how CSDaV infection progresses in host plants [5]. Although CSDaV was shown to be infectious to the model plant *N. benthamiana*, initial analyses presumed that the infection was restricted to the *Agrobacterium*-mediated inoculated leaves [5]. Thus, studies on CSDaV biology were limited to agroinoculated leaves, which rapidly (within 5 days post-inoculation) deteriorate due to severe hypersensitive response (HR)-like necrotic symptoms induced by the virus infection [5]. Here, we demonstrate that, contrary to previous observations, CSDaV is capable of establishing a symptomless systemic infection in *N. benthamiana*. This finding opens new possibilities for using this model system to investigate the infection cycle of CSDaV in detail, from local to systemic levels. In this study, we revisit the local infection site to uncover the cellular and molecular events underlying the virus–plant local interactions and their relationship to subsequent systemic virus movement. By investigating early virus replication sites, local cytological alterations and host responses in detail, we provide new insights into how CSDaV can induce severe local symptoms yet still achieve systemic infection.

## Methods

### Plants, viral infectious clones and expression constructs

*N. benthamiana* plants were grown and maintained in a growth chamber under a 16 h light/8 h dark photoperiod, at an average temperature of 22°C and 70% relative humidity

**Fig. 1.**
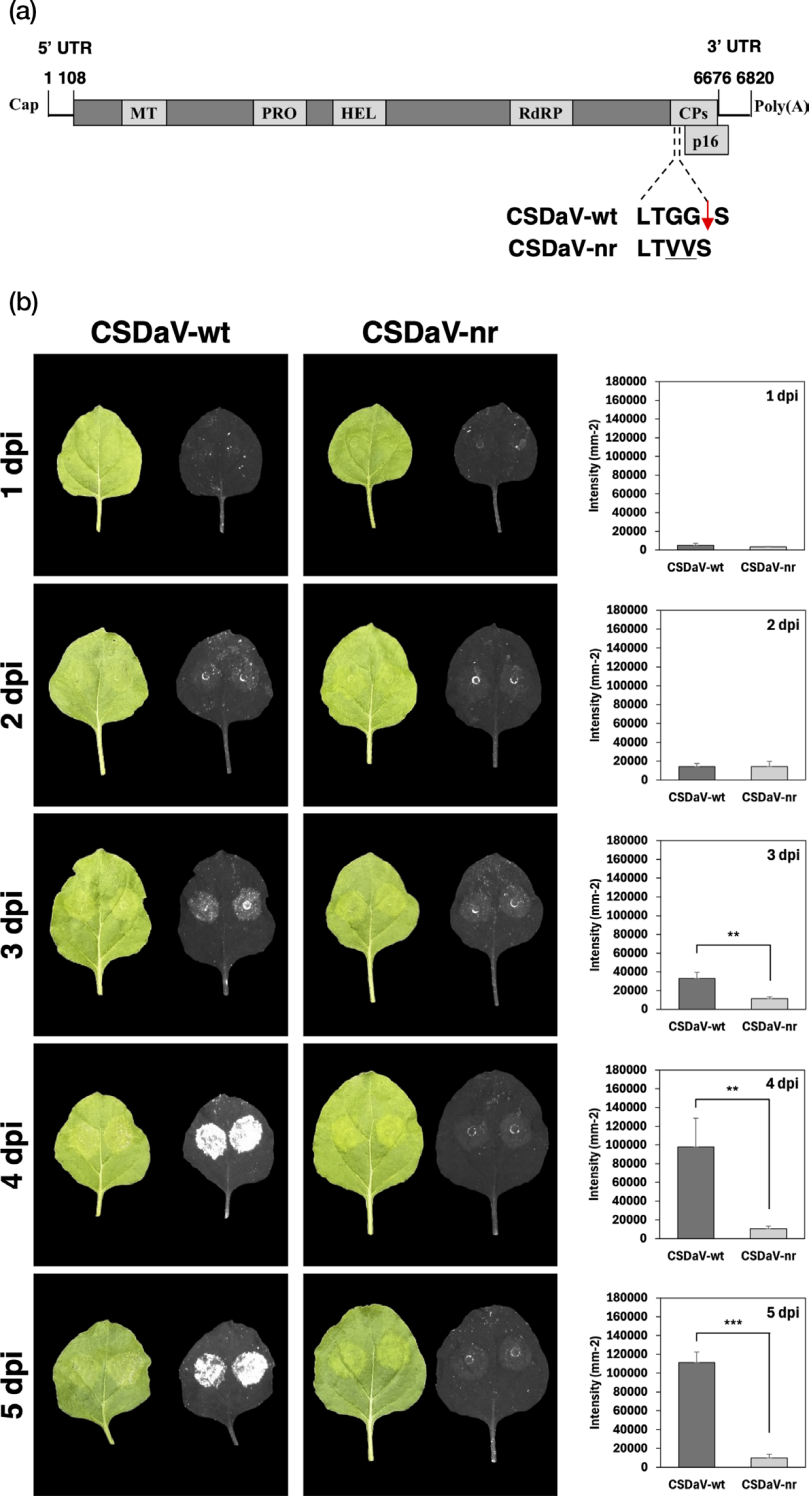
Time course of cell death development in *N. benthamiana* leaves infiltrated with *Agrobacterium tumefaciens* harbouring CSDaV wild-type (wt) or non-replicative (nr, negative control) clones. (a) Genomic map of CSDaV indicating the mutations (amino acid substitutions) introduced in the non-replicative viral clone, CSDaV-nr, used in this experiment. (b) Cell death was monitored daily from 1 to 5 days post-infiltration (dpi) using visible light imaging (left and middle panels, leaves on the left) and red light imaging (left and middle panels, leaves on the right). Representative images are shown. In the right panel, red light emission is expressed as intensity per square millimetre. Data represent the arithmetic mean of three biological replicates. The mean signal intensity of each biological replicate was calculated from two infiltrated areas. Statistical significance was determined using one-way ANOVA followed by Tukey’s multiple comparisons post hoc test (*P*≤0.05). Significant differences are indicated with asterisks: **indicates *P*≤0.01, and *** indicates *P*≤0.001.

The CSDaV clones used in this study [wild-type (CSDaV-wt) and non-replicative (CSDaV-nr)] were obtained from previously published work [5], where CSDaV-nr was named M-CS, referring to amino acid mutations in the cleavage site between the CSDaV RNA-dependent RNA polymerase (RdRP) and coat protein (CP) (Fig. 1a). The constructs for expressing the CSDaV RdRP were generated in this study. The genomic region encoding the RdRP was amplified by PCR from the CSDaV-wt infectious clone using Q5® High-Fidelity DNA Polymerase (New England Biolabs, USA), following the manufacturer’s protocol. Primers included attB sites for Gateway® cloning and start/stop codons to ensure proper protein translation (Table S1, available in the online Supplementary Material).

PCR products were purified using the Wizard® SV Gel and PCR Clean-Up System (Promega) before cloning into the Gateway™ pDONR™/Zeo vector (Invitrogen™, USA). Subsequently, the RdRP insert was transferred into the binary vectors pH7WGC2 and pH7CWG2 [15] to generate constructs with cyan fluorescent protein (CFP) fused to the N- (pH7WGC2-CFP-RdRP) or C-terminus (pH7CWG2-RdRP-CFP) of the CSDaV RdRP.

The resulting constructs were introduced into *Escherichia coli* DH10*β* cells via electroporation. Transformed cells were plated on Luria–Bertani (LB) agar containing the appropriate antibiotic (zeocin for pDONRZeo; spectinomycin for pH7WGC2 and pH7CWG2) and incubated overnight at 37°C. Colony PCR using M13F/M13R primer pair (Table S1) was performed to confirm plasmid presence. Plasmids were then purified using the Promega PureYield™ Plasmid Miniprep System (Promega, USA), and the inserted fragments were verified by Sanger sequencing.

Finally, the expression vectors were transformed into *Agrobacterium tumefaciens* strain GV3101 cells via electroporation. Transformed cells were grown for 2 days at 28°C in selected LB medium containing rifampicin and spectinomycin. Colony PCR was used to confirm the presence of the plasmids.

### *A. tumefaciens*-mediated infiltration

*A. tumefaciens* strain GV3103 cultures harbouring the desired constructs were prepared by first growing a pre-inoculum overnight at 28°C in liquid LB medium supplemented with the appropriate antibiotics. The following day, 50 µl of the pre-inoculum was transferred to 25 ml of LB containing 10 mM MES and 100 µM acetosyringone and incubated overnight at 28°C with shaking. When cultures reached an OD600 between 0.8 and 1.2, cells were harvested by centrifugation at 4700 r.p.m. for 10 min at 4°C and then resuspended in infiltration buffer (10 mM MES pH 5.6, 10 mM MgCl2 and 200 µM acetosyringone) to a final OD600 between 0.7 and 1.0 (depending on the experiment) for the CSDaV clones and 0.5 for the RdRP constructs. The suspension was incubated for at least 3 h at room temperature (RT) in the dark prior to infiltration. The bacterial suspension was infiltrated into the abaxial side of leaves of 3/4-week-old *N. benthamiana* plants using a needleless syringe with gentle pressure. When co-infiltration of two constructs was needed, the two suspensions, each harbouring one of the constructs, were mixed at a 1:1 ratio prior to infiltration.

### Symptom analysis by red light imaging

Cell death was quantified using the red light imaging system as described by Landeo Villanueva *et al*. [16]. Leaves were collected from *N. benthamiana* plants spot-infiltrated with CSDaV-wt or CSDaV-nr (negative control) clones at 1, 2, 3, 4 and 5 days post-infiltration (dpi). Images were acquired with a ChemiDoc MP Imaging system (Universal Hood III, Bio-Rad) using a Green Epi 595/95 excitation filter and a Red Epi 605/50 emission filter. The exposure time was optimized to avoid image saturation and set on 2 s. Images were processed using Image Lab software (version 6.0.1), and for comparison, reasons were set to the same image transform settings (high: 2500, low: 0 and gamma: 1.00). Mean signal intensity was calculated from three biological replicates, by manually selecting two infiltrated areas of each replicate and subtracting the background signal of two non-infiltrated areas of the same leaf. Red light emission was expressed as intensity per square millimetre. Statistical significance was determined using one-way ANOVA followed by Tukey’s multiple comparisons post hoc test (*P*≤0.05).

### Cytological analysis using light and electron microscopy

Leaves of *N. benthamiana* plants agroinfiltrated with the CSDaV-wt infectious clone, as well as leaves from a healthy control, were collected at 2 dpi, prior to the appearance of CSDaV-induced local symptoms. Small leaf pieces (~1–3 mm²) were excised using a razor blade and immediately fixed overnight at 4°C in Karnovsky’s fixative [2.5% (v/v) paraformaldehyde and 2% (v/v) glutaraldehyde in 0.08 M sodium phosphate buffer, pH 7.2]. Following fixation, samples were rinsed three times with 0.1 M sodium phosphate buffer (pH 7.2). Post-fixation was carried out in 1% osmium tetroxide in 0.1 M sodium phosphate buffer. Samples were then dehydrated through a graded ethanol series and embedded in a mixture of EPON and Spurr resin.

Cross-sections were prepared using a Leica EM UC6 ultramicrotome. For light microscopy, semithin sections (1.5 µm thick) were stained with 1% toluidine blue O in 1% sodium borate for 30 s, rinsed with distilled water, dried and mounted with coverslips. For transmission electron microscopy (TEM), ultrathin sections (~100 nm thick) were collected on copper grids and stained with uranyl acetate and lead citrate. Grids were examined using a JEOL 2100F transmission electron microscope (JEOL USA Inc., Peabody, MA, USA) operated at an accelerating voltage of 200 kV.

### Immunogold localization

Leaves from both healthy control and CSDaV-wt agroinfiltrated *N. benthamiana* plants were collected at 2 dpi. Leaf samples were cut into ~1–3 mm² pieces and fixed overnight at 4°C in 4% paraformaldehyde prepared in 0.1 M sodium phosphate buffer (pH 7.2). After fixation, samples were rinsed three times with 0.1 M sodium phosphate buffer and incubated in 0.1% tannic acid for 30 min, followed by three rinses with cold distilled water. Tissues were dehydrated through a graded ethanol series and subsequently incubated on ice in 2% uranyl acetate in 50% ethanol for 1 h. Samples were then rinsed 3 x with 70% ethanol, followed by 3 x with 95% ethanol and 2× with 100% ethanol for 20 min each. Samples were then embedded in LR white resin for thin sectioning, following standard procedures [17].

Ultrathin sections were mounted on formvar/carbon-coated nickel grids (TED PELLA, #01800 N-F). Grids were blocked in a drop of blocking buffer (1% BSA, 10 mM Tris-HCl pH 7.4, 100 mM NaCl and 0.1% Tween-20) for 10 min and then incubated for 1 h at RT in a drop of anti-CSDaV CP antibody (custom-synthesized by Genescript based on peptide C-GPAPSRDDRVDRQP) diluted 1:200. After five washes with Tris-EDTA (TE) buffer, grids were re-blocked for 10 min and incubated for 1 h at RT with a 1:30 dilution of secondary antibody conjugated to 10 nm gold particles (goat anti-rabbit; TED PELLA, #15726). Grids were then washed five times with TE buffer and post-stained with 1% uranyl acetate prior to examination by TEM.

### Western blot analysis

Leaf discs were collected at 6 dpi from local *N. benthamiana* leaves transiently co-expressing CFP-RdRP or RdRP-CFP with the non-replicative CSDaV-nr clone. Discs from leaves co-infiltrated with either the CSDaV-nr or CSDaV-wt clone and an empty vector were also collected as controls.

Collected leaf tissue (100 mg) was ground in 300 µl of protein extraction buffer [5 mM DTT, 50 mM NaCl, 100 mM EDTA, 100 mM Tris-HCl (pH 7.5) and 0.1% Triton X-100]. Samples were incubated at RT for 5 min with shaking, then centrifuged at 12,000 r.p.m. for 10 min at 4°C. Fifteen microlitres of the protein extract (supernatant) was mixed with 15 µl of protein loading buffer [100 mM Tris (pH 7.5), 200 mM DTT, 0.2% bromophenol blue, 2% glycerol, 4% SDS and 10% *β*-mercaptoethanol] and heated at 95°C for 10 min. Proteins were separated by SDS-PAGE using a 12.5% precast gel (Bio-Rad, 456-9033) in Tris/glycine buffer (25 mM Tris-HCl, 250 mM glycine and 0.1% SDS) and transferred to a PVDF membrane using a power blotter (ThermoFisher, PB0012; 25 V, 2.5 A, 10 min). The Coomassie Blue-stained ribulose-1,5-bisphosphate carboxylase/oxygenase large subunit was used as a loading control for the total proteins.

The PVDF membrane was blocked with 1% milk in Tris-buffered saline with Tween (TBS-T, 20 mM Tris, 150 mM NaCl and 0.1% Tween-20) overnight at 4°C and then incubated with a 1:2,000 dilution of rabbit anti-CSDaV CP antibody for 1 h at RT. An alkaline phosphatase-conjugated goat anti-rabbit polyclonal antibody (Dako, D0487) was used as the secondary antibody. Blots were developed using a 1:100 dilution of NBT/BCIP solution (Roche, 11681451001) in alkaline phosphatase buffer (5 mM MgCl₂, 100 mM NaCl and 100 mM Tris-HCl, pH 9.5).

### Confocal microscopy

To visualize the subcellular localization of the CSDaV RdRP during the rescue of a replication-deficient CSDaV clone, *N. benthamiana* leaves singly infiltrated with RdRP-CFP or co-infiltrated with the non-replicative CSDaV-nr clone were examined for CFP fluorescence and chlorophyll autofluorescence using a confocal laser scanning microscope (Leica Stellaris 5) equipped with an HC PL APO CS2 20×/0.75 DRY objective (Leica Systems, 11506517).

Leaf tissue was checked at 6 dpi. Samples were mounted on microscope slides by placing a 0.5×0.5 cm leaf section with the abaxial side facing up to observe epidermal cells. Fluorescence was detected using excitation at 440 nm, with emission collected at 450–495 nm for CFP and at 652 nm for chlorophyll autofluorescence.

### CSDaV detection in upper, non-inoculated leaves

To assess the ability of CSDaV to systemically infect *N. benthamiana*, plants were agroinfiltrated with either the CSDaV-wt infectious clone or CSDaV-nr (used as a negative control). Leaf discs were collected from upper non-inoculated leaves at 23 dpi. Systemic viral infection was evaluated by strand-specific reverse transcription polymerase chain reaction (RT-PCR) (for both positive and negative strands) and by Western blot analysis for detection of CSDaV CPs.

For strand-specific RT-PCR, total RNA was extracted using TRIzol Reagent (Invitrogen, 15596026) according to the manufacturer’s instructions. RNA was reverse transcribed using M-MLV reverse transcriptase (Promega, M170B), following the manufacturer’s protocol. For positive-strand detection, random primers (Roche, 11034731001) were used; for negative-strand detection, a strand-specific primer (Table S1) was used. PCR amplification targeted the RdRP genomic region for both positive- and negative-strand detection (Table S1). For the detection of the CSDaV CPs by Western blot, systemic leaves were processed as described in the Western blot analysis section.

### Measurement of total reactive oxygen species content

Total reactive oxygen species (ROS) content was measured using 2′,7′-dichlorodihydrofluorescein diacetate (DCFDA) (Sigma-Aldrich, D6883), following the protocol described by Jambunathan [18]. Two leaf discs were collected from local *N. benthamiana* leaves agroinfiltrated with either the CSDaV-wt infectious clone or CSDaV-nr (used as a negative control) at 0 dpi and at 12, 24, 48 and 72 h post-infiltration (hpi). Immediately after harvesting, samples were submerged in liquid nitrogen until processing.

Approximately 35 mg of leaf tissue was ground in 350 µl of 10 mM Tris-HCl (pH 7.2), followed by centrifugation at 15,000 ***g*** for 20 min at 4°C. For ROS detection, 20 µl of the resulting leaf extract was mixed with 180 µl of Tris-HCl and 2 µl of 1 mM DCFDA. A parallel reaction without DCFDA (20 µl leaf extract+180 µl Tris-HCl) was included to assess background fluorescence. Samples were vortexed, transferred to a black polypropylene 96-well plate (Greiner Bio-One, 655209) and incubated at RT in the dark for 20 min. Relative fluorescence intensity was measured using a FLUOstar OPTIMA microplate reader (BMG LABTECH) with excitation at 485 nm, emission at 520 nm and gain set to 1,800.

The remaining supernatant was stored at −20°C for protein quantification using the Pierce™ BCA Protein Assay Kit (Thermo Scientific, 23227), according to the microplate protocol in the manufacturer’s manual. Standard solutions and twofold serial dilutions of each sample were prepared in 10 mM Tris-HCl (pH 7.2). To each 25 µl of standard or diluted sample, 200 µl of working reagent was added in a non-binding 96-well microplate (Greiner Bio-One, 655901) and incubated at 37°C for 30 min. Absorbance at 540 nm was measured in three technical replicates using the FLUOstar OPTIMA microplate reader. Protein concentrations were interpolated from a four-parameter logistic standard curve based on eight concentration points. For each sample, the mean fluorescence intensity (after subtraction of background signal) from three technical replicates was normalized to the total protein content. ROS levels were expressed as relative fluorescence units per microgramme of protein.

### Relative gene expression analysis

To quantify the accumulation of CSDaV genome copy number, as well as the expression of a ROS-related gene and genes involved in plant defence responses, reverse transcription-quantitative polymerase chain reaction was performed using the PowerTrack SYBR Green Master Mix (Applied Biosystems, A46109).

CSDaV RNA accumulation in infiltrated *N. benthamiana* leaves was quantified using primers specific for the RdRp gene (Table S1). CSDaV-wt plasmids were used to generate a standard curve. A 10-fold serial dilution ranging from 0.003 pg µl^−1^ to 30 ng µl^−1^ of purified plasmid was obtained. Plasmid copy numbers were calculated according to Plumet and Gerlier [19]. Viral copy numbers were interpolated from a standard curve assayed on each plate.

For the relative gene expression analysis, *protein phosphatase 2a* (*PP2a*) was used as a reference gene. The target genes *catalase 1* (*CAT1*), *hairpin-induced 1* (*HIN1*), *pathogenesis-related protein 1* (*PR1*) and *isochorismate synthase 1* (*ICS1*) were normalized to the *PP2a* reference gene expression levels. No template and no reverse transcriptase controls were also assayed for each target gene. Relative gene expression levels were calculated over CSDaV-nr as the negative control group, according to the ΔΔCq method described by Pfaffl [20]. The amplification efficiency (*E*) for each primer pair was calculated according to the equation *E*=10–(1/slope) and determined over a range from 0.063 to 2 ng µl^−1^ cDNA in total reaction volume. All reactions, including the reference gene *PP2a*, were performed with two technical replicates per biological sample. Five biological replicates were included in all cases.

## Results

### CSDaV infection induces a local HR-like reaction in *N. benthamiana* plants which is preceded by cytological abnormalities in chloroplasts

In a previous study, we reported that CSDaV locally infects *N. benthamiana* plants via *A. tumefaciens*-mediated inoculation of its infectious clone and induces a quick (within 3–5 dpi) and severe necrotic response on the agroinfiltrated leaves [5]. Because previous analyses were only based on visual cell death symptoms, here we performed a symptom analysis using a red light imaging system [16] to relatively quantify the amount of cell death induced by CSDaV in a more sensitive and objective manner. As expected, *N. benthamiana* leaves spot-infiltrated with the wild-type CSDaV infectious clone (CSDaV-wt) showed a clear increase in red light signal over time (starting from 3 dpi), indicating that the extent of cell death increases during CSDaV local infection (Fig. 1b, left panel). No red light signal was detected in leaves spot-infiltrated with the non-replicative CSDaV (CSDaV-nr) negative control (Fig. 1b, middle panel). At 3, 4 and 5 dpi, cell death induced by CSDaV-wt was statistically significantly higher compared to CSDaV-nr (*P*=0.0039, 0.0080 and 0.0001, respectively; Fig. 1b, right panel). The CSDaV genome copy number across the tested time points is shown in Fig. S1.

**Fig. 2.**
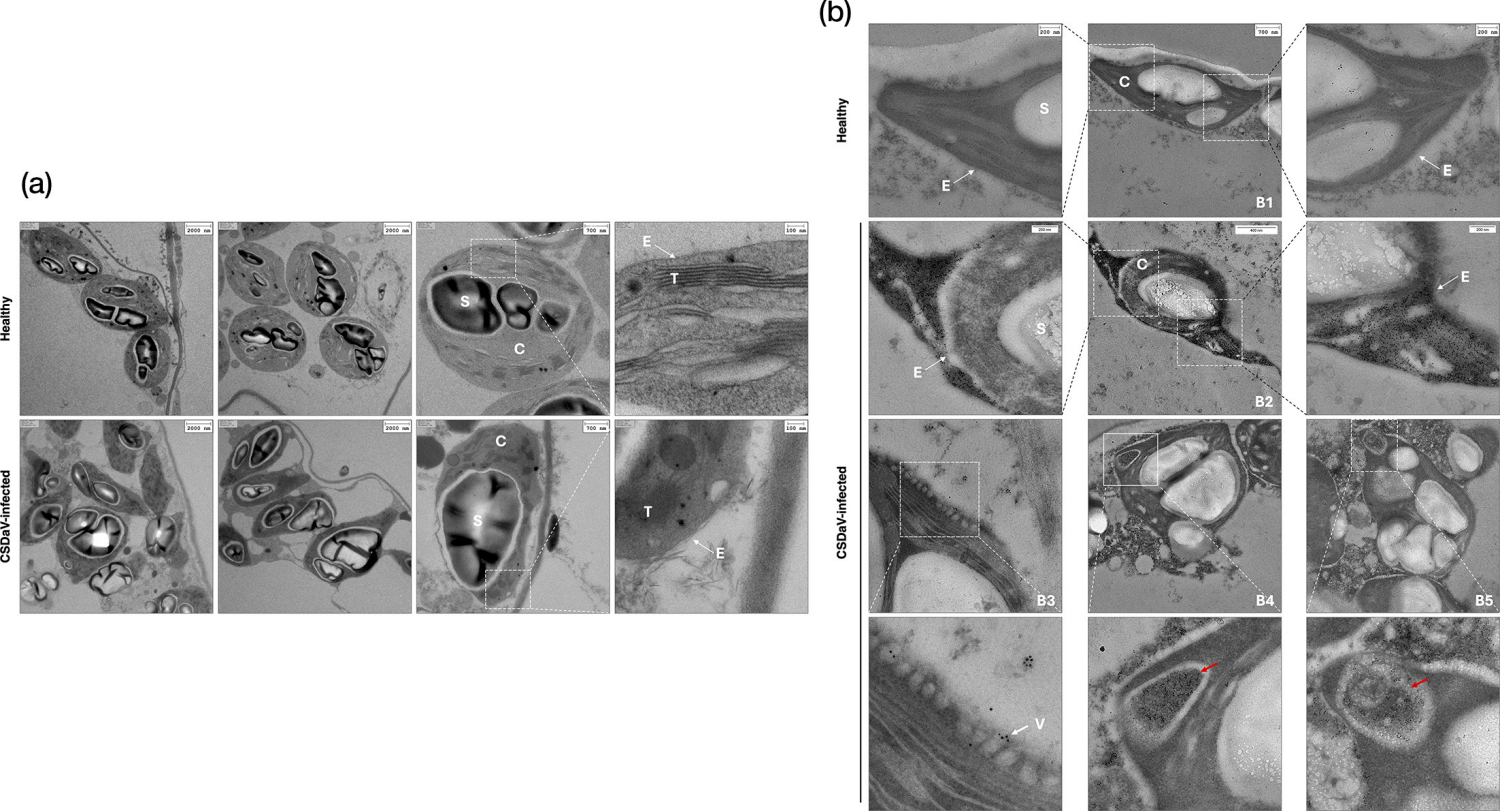
(a) Transmission electron micrographs of sections from healthy (top) and CSDaV-infected (bottom) *N. benthamiana* leaves. Dashed white squares on the chloroplast images in the third column (from left to right) indicate the regions enlarged in the fourth column. (b) Transmission electron micrographs of sections from healthy (B1) and CSDaV-infected (B2-5) *N. benthamiana* leaves, immunogold-labelled with antibody against CSDaV CPs. Dashed white squares on B1 and B2 indicate the regions enlarged on the left and right sides. Dashed white squares on B3-5 indicate the regions enlarged below each image. C, chloroplast; S, starch; T, thylakoids; E, envelope membranes; V, vesicles. Red arrows indicate gold particle aggregation inside chloroplast structures.

Next, we investigated whether CSDaV induces any cytological changes in the agroinfiltrated leaves. At 2 dpi, before the development of local symptoms, tissue samples from *N. benthamiana* leaves agroinfiltrated with CSDaV-wt infectious clone as well as from non-infiltrated (healthy) plants were processed for examination by light and electron microscopy. By light microscopy, we observed that the toluidine blue-stained semithin sections of CSDaV-infected leaves showed more chloroplast clumping compared to the semithin sections of healthy leaves (Fig. S2). When these leaves were examined by TEM, we observed clear abnormalities in chloroplast ultrastructure in thin sections of CSDaV-infected leaves compared to those of healthy leaves. In the infected tissue, chloroplasts exhibited irregular shapes; some were degraded, contained larger starch granules in relation to chloroplast size and had broken chloroplast envelopes with disrupted membrane (Fig. 2a).

To investigate the relationship between altered chloroplasts and CSDaV, we performed immunogold labelling using antibodies against the CSDaV CPs on CSDaV-infected tissues. In infected cells, immunolocalization revealed a higher density of gold particles around the altered chloroplasts, particularly in the cytosol near what appears to be a disrupted chloroplast envelope (Fig. 2-B2). Additionally, vesicle formation (Fig. 2-B3) and the presence of CSDaV particles inside chloroplasts (Fig. 2-B4 and B5) were observed in a few electron micrographs obtained in the present study. The specificity of the labelling was confirmed by performing the same procedure on non-infected tissue, which showed no specific labelling (Fig. 2-B1). These results suggest that the regions surrounding altered chloroplasts, or even internal chloroplast structures, may serve as sites of CSDaV replication and/or virion assembly.

### Functional CSDaV RdRP aggregates along cell boundaries during rescue of a replication-deficient CSDaV clone

**Fig. 3.**
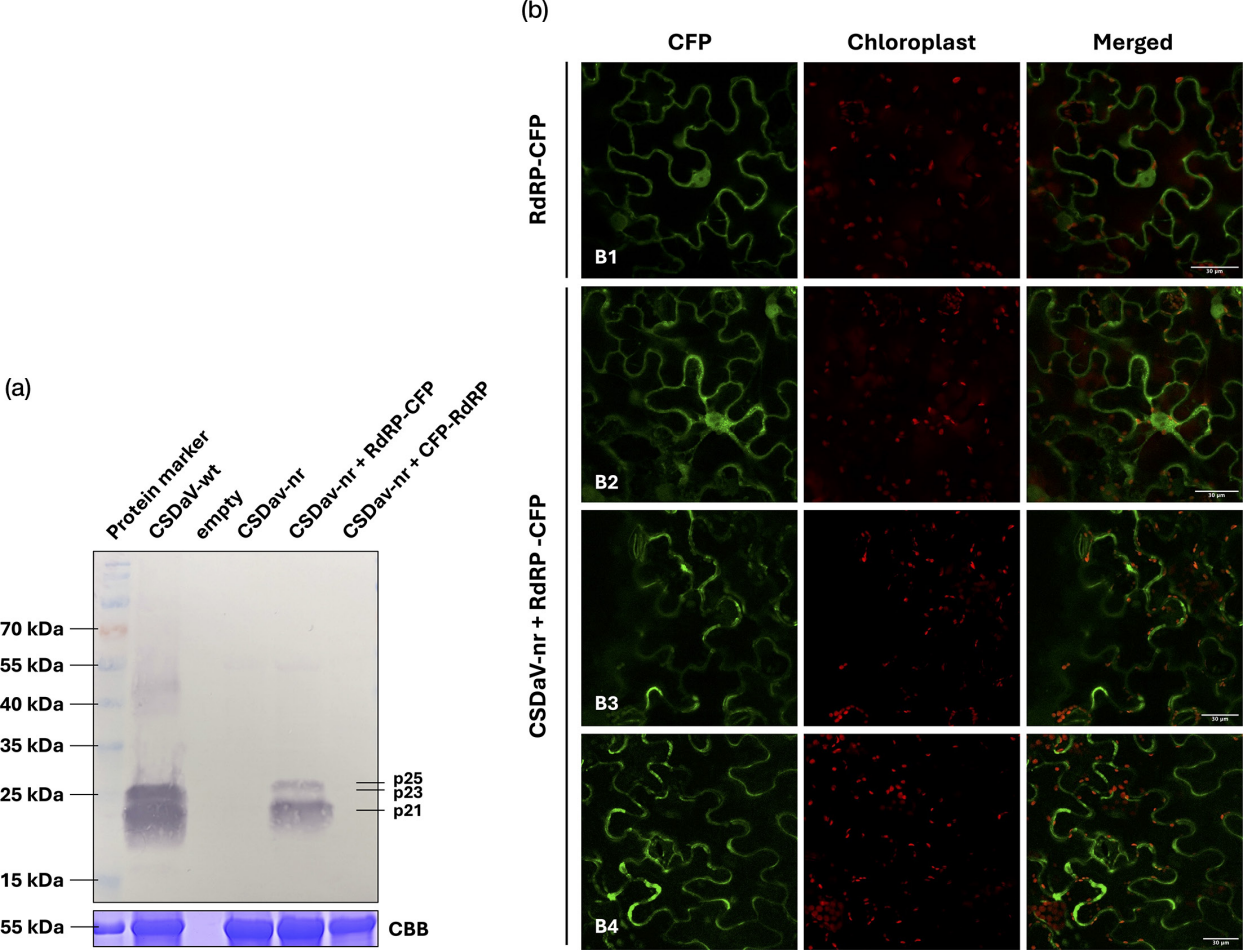
Trans-complementation of a replication-deficient CSDaV clone by the CSDaV RdRP protein. (a) Western blot analysis of total proteins extracted from *N. benthamiana* leaves infiltrated with *A. tumefaciens* harbouring CSDaV wild-type (wt) or non-replicative (nr, negative control) clones, or co-infiltrated with CSDaV-nr and CSDaV RdRP either N- or C-terminally fused to CFP. Successful trans-complementation was evaluated by detection of CSDaV quasi-equivalent CPs (p25, p23 and p21), which are produced from subgenomic RNA upon virus replication. Ribulose-1,5-bisphosphate carboxylase/oxygenase (Rubisco) Coomassie blue (CBB) staining is shown as loading control. (b) Subcellular localization of the RdRP-CFP fusion protein during trans-complementation of CSDaV-nr replication was assessed by confocal microscopy. Localization of RdRP-CFP expressed alone is shown as a control.

To gain further insight into where CSDaV replicates in infected cells, we first attempted to determine whether the CSDaV RdRP could rescue, in trans, replication of the replication-deficient CSDaV-nr clone. To test this, we co-infiltrated *N. benthamiana* leaves with *A. tumefaciens* strains carrying the CSDaV-nr construct (previously made and described in [5]) and a construct encoding the viral RdRP fused to CFP, either at its N- or C-terminus. Western blot analysis of leaf tissue collected at 6 dpi revealed the presence of the viral CPs only when the CSDaV-nr clone was co-expressed with the C-terminal RdRP-CFP fusion, but not with the N-terminal CFP-RdRP fusion (Fig. 3a). No CPs were detected in leaves infiltrated with the CSDaV-nr construct alone. Because the three CSDaV CPs (p25, p23 and p21) are produced from a subgenomic RNA transcribed from an internal promoter on the viral negative strand [5], its expression serves as an indicator of active viral replication. Although the minor CPs (p25 and p23) could not be distinguished individually on the blot, one or both were detected alongside the major CP (p21). The absence of CPs in leaves infiltrated with CSDaV-nr alone and their presence in samples co-infiltrated with RdRP-CFP, respectively, confirm the inability of CSDaV-nr to replicate autonomously and demonstrate successful replication complementation by the RdRP-CFP fusion construct.

To further investigate the subcellular localization of the trans-complemented CSDaV replication machinery, we examined leaf tissues co-infiltrated with CSDaV-nr and RdRP-CFP by confocal microscopy. Fluorescence from RdRP-CFP displayed a uniform cytoplasmic and nuclear distribution when expressed individually (Fig. 3-B1); however, it was redistributed into less uniform aggregates along the cell boundaries under conditions in which viral replication was rescued (Fig. 3-B3 and B4). No clear chloroplast association of the viral replication sites was observed, although chloroplasts in leaves infiltrated with the RdRP-CFP only appeared regularly spaced and mostly confined to the cell periphery, whereas in the virus-replicating context, chloroplasts were more clustered (also around the cell nucleus, Fig. 2-B2), suggesting virus-induced structural rearrangements or chloroplast repositioning.

### The HR-like reaction induced by CSDaV local infection does not restrict systemic viral movement

**Fig. 4.**
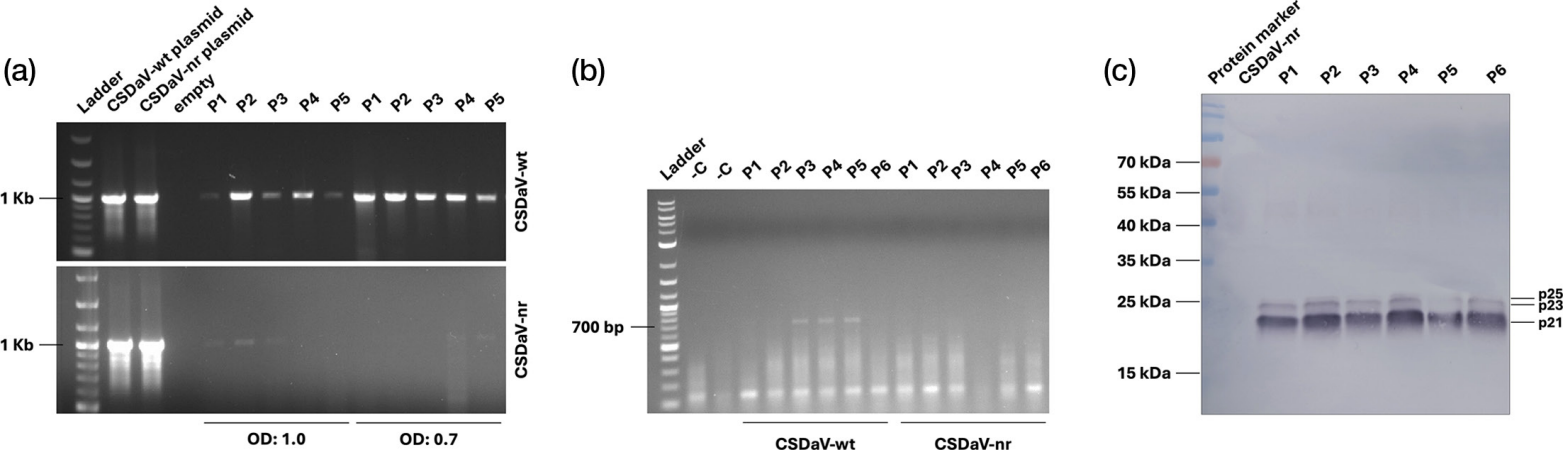
Detection of CSDaV in upper, non-inoculated leaves of *N. benthamiana* plants at 23 days post-inoculation via *A. tumefaciens* carrying CSDaV wild-type (wt) or non-replicative (nr; negative control) clones. (a) RT-PCR detection of CSDaV-positive strand RNA. Expected PCR product size: 1,036 bp. (b) RT-PCR detection of CSDaV-negative-strand RNA. Expected PCR product size: 694 bp. (c) Immunoblot detection of CSDaV by using the anti-CSDaV CP antibody. Expected molecular weights of CSDaV CPs: 21, 23 and 25 kDa. P1–P6, individual plant replicates per treatment; OD, OD of the *Agrobacterium* suspension used for infiltration. –C, healthy plant control.

Since HR responses to virus infections in plants normally restrict the virus to local infection sites, and no symptoms were observed in upper leaves of *N. benthamiana* plants agroinfiltrated with the CSDaV infectious clone, we initially presumed that CSDaV was unable to systemically infect *N. benthamiana* plants [5]. However, to unequivocally determine if CSDaV was restricted to inoculated leaves, we tested upper leaves from agroinoculated plants. CSDaV was detected in the upper leaves of the 10 CSDaV-wt-infiltrated plants, when they were tested at 23 dpi (Fig. 4a, top). Moreover, follow-up experiments showed that the CSDaV could already be detected in upper, non-inoculated leaves as early as 7 or 14 dpi (Fig. S3). However, because RT-PCR detection of CSDaV in upper leaves unexpectedly resulted in faint bands for a few (five out of ten) plants agroinfiltrated with the CSDaV-nr control (Fig. 4a, bottom), we further attempted to detect CSDaV in the upper leaves by performing both negative-strand-specific RT-PCR- and Western blotting-based detections. The negative-strand-specific RT-PCR assay yielded clear products of the expected size in upper leaves of three out of six plants agroinfiltrated with CSDaV-wt infectious clone, whereas no RT-PCR products were observed in upper leaves of CSDaV-nr-infiltrated plants (Fig. 4b), which suggests viral replication. Additionally, CSDaV CPs were detected in upper leaves of all six tested plants by using an antibody against its CPs in a Western blotting (Fig. 4c), further confirming systemic CSDaV movement. Still, no systemic symptoms were observed in CSDaV-infected plants (Fig. S4).

### CSDaV triggers local HR-like reaction but likely suppresses systemic resistance

Since the HR-like reaction induced by CSDaV local infection did not restrict systemic viral movement, we further investigated the plant’s local response to CSDaV infection in more detail. Specifically, we examined hallmark features of the HR, including the expression of *hairpin-induced 1* (*HIN1*), a well-known HR marker gene; the accumulation of ROS; and the expression of *catalase 1* (*CAT1*), an antioxidant gene.

**Fig. 5.**
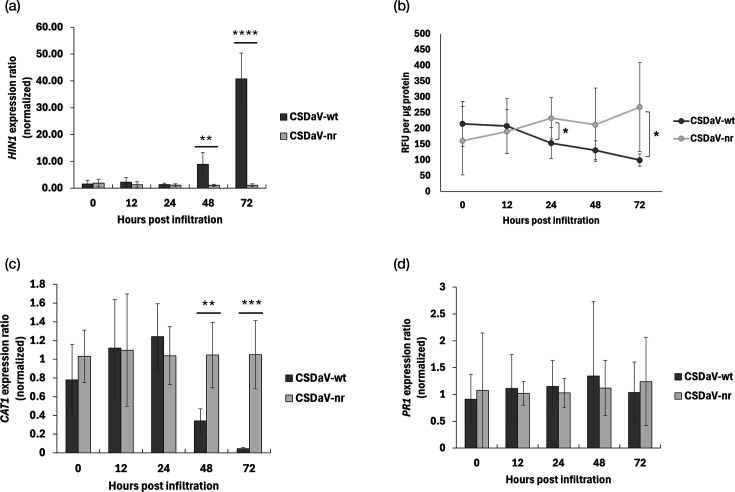
Local responses to CSDaV infection in *N. benthamiana* leaves infiltrated with *A. tumefaciens* harbouring CSDaV wild-type (wt) or non-replicative (nr, negative control) clones. (a) Expression levels of *hairpin-induced 1* (*HIN1*), a marker for HR. (b) Total ROS content. (c) Expression levels of *catalase 1* (*CAT1*), an ROS-scavenging gene. (d) Expression levels of *pathogenesis-related protein 1* (*PR1*), a marker for systemic acquired resistance (SAR). Analyses were performed at 0, 12, 24, 48 and 72 h post-infiltration. In the case of gene expression analysis, *PP2a* was used as a reference gene for normalization. In the case of ROS measurements, data are expressed as relative fluorescence units per microgramme protein. For each time point, data were tested for significant differences (*P*≤0.05) using the appropriate unpaired t-test according to data being normally distributed and the homogeneity of variances. Significant differences are indicated with asterisks: * indicates *P*≤0.05, ** indicates *P*≤0.01, and *** indicates *P*≤0.001.

*HIN1* expression was significantly upregulated in *N. benthamia*na leaves at 48 and 72 h post-agroinfiltration (hpi) with the CSDaV-wt infectious clone, compared to leaves infiltrated with the CSDaV-nr control clone (Fig. 5a), indicating activation of an HR-like response. Surprisingly, total ROS levels were consistently lower in CSDaV-wt-infiltrated leaves compared to the control at 24, 48 and 72 hpi, with statistically significant differences observed at 24 and 72 hpi (Fig. 5b). Correspondingly, the expression of *CAT1*, which encodes an antioxidant enzyme typically induced in response to ROS accumulation, to detoxify H₂O₂, was significantly downregulated at 48 and 72 hpi in CSDaV-wt-infiltrated leaves compared to the control (Fig. 5c). These results suggest that CSDaV may trigger an HR-like response in *N. benthamiana* leaves that is independent of ROS accumulation.

Additionally, we assessed the expression of *pathogenesis-related protein 1* (*PR1*), a marker of systemic acquired resistance (SAR). No significant differences in *PR1* expression were observed at any of the time points analysed between plants agroinfiltrated with CSDaV-wt and those with CSDaV-nr (Fig. 5d), suggesting that SAR is not prominently engaged in the response to CSDaV infection. Since salicylic acid (SA) is the key phytohormone involved in SAR signalling and is synthesized in chloroplasts, an organelle likely affected by CSDaV infection, we also analysed the expression of the *isochorismate synthase 1* (*ICS1*) gene, which catalyses a key step in SA biosynthesis from chorismic acid in chloroplasts. At 48 and 96 hpi, *ICS1* expression was significantly downregulated in leaves infiltrated with the CSDaV-wt infectious clone compared to the CSDaV-nr control (Fig. S5). This result suggests that CSDaV may suppress SA biosynthesis by repressing *ICS1*, thereby impairing systemic signalling and SA-dependent defence responses.

## Discussion

In this study, we revisited the interaction between CSDaV and its non-natural host *N. benthamiana*, a widely used experimental model plant for studying virus-plant interactions. Our current results, based on infections initiated by the CSDaV infectious clone, contradict previous assumptions that CSDaV infection in *N. benthamiana* is strictly limited to locally agroinoculated leaves [5]. We demonstrate that, despite the virus inducing a strong HR-like reaction at the site of inoculation, CSDaV is capable of moving systemically and establishing an asymptomatic infection in distal tissues. This result not only advances our understanding of CSDaV biology in this model host but also provides a refined experimental system for dissecting CSDaV infection at the local and systemic levels. Here, we focused on characterizing the CSDaV infection at the local infection site.

Red light imaging and gene expression analyses confirmed that CSDaV induces rapid local cell death associated with hallmark features of HR. This response was dependent on viral replication, as a replication-deficient clone (CSDaV-nr) failed to elicit necrosis or detectable red light signals. Classically, HR is viewed as an effective antiviral response that restricts viral infection to the initially infected area through localized programmed cell death [21]. However, despite the local HR-like response, CSDaV was able to move systemically within the plant. Viral RNA and CPs were detected in upper, non-inoculated leaves as early as 7 dpi and consistently by 23 dpi, demonstrating systemic movement. Detection of the CSDaV-negative RNA strand and CPs in these tissues – by strand-specific RT-PCR and Western blotting, respectively – confirmed active replication. A similar phenomenon has been observed in some other plant–virus systems. For instance, a potato virus X (PVX; *Potexvirus ecspotati*) vector expressing the replication-associated protein (Rep) of two distinct begomoviruses induced a local HR-like response in inoculated *N. benthamiana* leaves, which did not prevent the systemic infection of the recombinant viruses [22]. The authors speculated that the host local response specifically targets Rep after its translation and relocation to the nucleus, thereby allowing PVX sufficient time to replicate and move away from the initially infected cells before the infection is restricted in necrotic tissues [22]. This suggests that CSDaV replication and movement may outpace the spread of the local HR-like response signal. However, PVX expressing Rep also induced severe systemic necrosis in inoculated plants [22], whereas CSDaV systemically infected tissues showed no visible symptoms, indicating that CSDaV may establish a latent or asymptomatic infection beyond the initially inoculated leaves. Similar observations were reported for the recently described wheat umbra-like virus infectious clone, where agroinfiltrated leaves developed severe necrosis, but upper, infected leaves remained symptomless [23].

Another key observation in this study is that the HR-like reaction induced by local CSDaV infection is preceded by cytological abnormalities in chloroplasts, including clumping, disrupted envelope membranes and the accumulation of large starch granules, suggesting an early organelle dysfunction associated with CSDaV infection. Moreover, our immunogold labelling experiments detected the presence of CSDaV CPs near damaged chloroplasts, suggesting that these organelles (or their surrounding cytoplasm) may serve as replication-associated or virion assembly sites. Using a complementation assay with a CFP-tagged RdRP, we demonstrated that replication of the non-replicating CSDaV clone can be rescued by the C-terminal fusion RdRP-CFP, but not by the N-terminal fusion (CFP-RdRP). Recovery of CP expression in this system confirmed that the RdRP retains its functionality when fused at the C-terminus and that CSDaV replication can be reconstituted in trans. Confocal microscopy showed that RdRP-CFP within the trans-complemented system re-localized into aggregates at the cell boundaries, suggesting potential sites of CSDaV replication, but no co-localization or clear association with chloroplasts was observed. Nevertheless, chloroplasts in leaves infiltrated with RdRP-CFP alone appeared regularly spaced and mostly confined to the cell periphery, whereas in the virus-replicating context, chloroplasts were more clustered (also around the cell nucleus), suggesting virus-induced structural rearrangements or chloroplast repositioning. These observations are consistent with increasing evidence that chloroplasts are directly targeted or indirectly affected by diverse plant viruses and undergo major structural and functional changes during infection [24,25]. Relevant examples include members of the genus *Tymovirus* within the family *Tymoviridae*, which induce infections characterized by a distinctive chloroplast cytopathology [26]. The most extensively studied virus within this genus is turnip yellow mosaic virus (TYMV; *Tymovirus brassicae*). Its 140K replication protein is a key organizer of the viral replication complex (VRC), being responsible for recruiting the viral polymerase and targeting VRCs to the chloroplast envelope where replication occurs [27]. This protein also induces chloroplast clumping, one of the hallmark cytological effects of TYMV infection, in addition to other abnormalities such as swelling and vesicle formation [28]. In fact, although the trans-complementation assay with RdRP-CFP did not clearly support CSDaV replication in chloroplasts, vesicular invaginations at chloroplast membranes and the presence of CSDaV CPs/particles inside chloroplasts were observed in a few electron micrographs obtained in the present study, strongly suggesting chloroplast-associated replication. Thus, it is possible that the trans-complemented CSDaV replication assay does not fully behave like the wild-type virus. Therefore, determining whether and how chloroplasts are involved with CSDaV replication or whether its replication protein is involved in chloroplast targeting similarly to TYMV still requires further investigation.

To investigate the local response to CSDaV infection, we examined molecular markers associated with plant defence. While *HIN1*, an HR-associated gene, was upregulated, total ROS levels were significantly reduced in CSDaV-infected tissues compared to the negative control. This reduction was accompanied by downregulation of *CAT1*, a gene encoding a ROS-scavenging enzyme, suggesting that CSDaV may actively suppress ROS accumulation, perhaps to avoid triggering stronger defence responses. Given that ROS bursts are generally associated with HR and plant resistance [29], these results suggest that CSDaV may induce an ROS-independent HR-like response. Similar observations have been reported for other plant pathogens. For instance, Yano *et al*. [30] demonstrated that ROS accumulation is not required for the cell death response triggered by the fungal pathogen *Trichoderma viride* (TvX), suggesting the involvement of a ROS-independent signalling pathway. Additionally, Zurbriggen *et al*. [31] reviewed cases in which ROS production is abolished while aspects of the HR remain unaffected, indicating that ROS-independent processes also contribute to HR. However, as far as is known, this has not been reported in the context of plant viral infections.

Furthermore, CSDaV infection did not lead to significant upregulation of *PR1*, a marker of SAR, and instead repressed the expression of *ICS1*, a key gene involved in SA biosynthesis in chloroplasts. Since SA, synthesized in chloroplasts, is central to SAR, this repression implies that CSDaV interferes with SA-mediated defence early in infection. Whether the observed chloroplast dysfunction in CSDaV-infected cells may directly impair SA production is not known, but if so, it might contribute to reduced systemic signalling and enabling CSDaV to bypass local defence response to successfully infect the plant systemically.

Altogether, our findings reveal that CSDaV elicits an HR-like response in *N. benthamiana* plants that is characterized by early chloroplast alterations, limited ROS accumulation and a likely suppression of SA signalling. However, further analyses measuring SA concentrations in CSDaV-infected plants are needed to validate this conclusion. Despite the local response, CSDaV is still able to spread and replicate systemically, indicating that systemic host defences are either insufficient, counteracted by viral mechanisms, or that HR-based defence responses do not occur once the virus reaches the phloem. Our model system provides a tractable platform to investigate CSDaV replication, movement and host manipulation at both cellular and systemic levels. Moreover, the ability to decouple local HR-like responses from systemic immunity in this system may offer the opportunity to gain broader insights into viral strategies for immune evasion.

## Supplementary material

10.1099/jgv.0.002252 Uncited Supplementary Material 1.

## Abbreviations

CFP: cyan fluorescent protein
CP: coat protein
CSD: citrus sudden death
CSDaV: citrus sudden death-associated virus
CTV: citrus tristeza virus
DCFDA: 2′,7′-dichlorodihydrofluorescein diacetate
dpi: days post-infiltration
HR: hypersensitive response
LB: Luria–Bertani
PVX: potato virus X
RdRP: RNA-dependent RNA polymerase
ROS: reactive oxygen species
RT: room temperature
SA: salicylic acid
SAR: systemic acquired resistance
TE: Tris-EDTA
TEM: transmission electron microscopy
TYMV: turnip yellow mosaic virus
VRC: viral replication complex

## References

[1] Brewer HC, Hird DL, Bailey AM, Seal SE, Foster GD (2018). A guide to the contained use of plant virus infectious clones. Plant Biotechnol J.

[2] Sanfaçon H (2017). Grand challenge in plant virology: understanding the impact of plant viruses in model plants, in agricultural crops, and in complex ecosystems. Front Microbiol.

[3] Ambrós S, El-Mohtar C, Ruiz-Ruiz S, Peña L, Guerri J (2011). Agroinoculation of *Citrus tristeza* virus causes systemic infection and symptoms in the presumed nonhost *Nicotiana benthamiana*. Mol Plant Microbe Interact.

[4] Huang Q, Hartung JS (2001). Cloning and sequence analysis of an infectious clone of *Citrus* yellow mosaic virus that can infect sweet orange via *Agrobacterium*-mediated inoculation. J Gen Virol.

[5] Matsumura EE, Coletta-Filho HD, Machado MA, Nouri S, Falk BW (2019). Rescue of *Citrus* sudden death-associated virus in *Nicotiana benthamiana* plants from cloned cDNA: insights into mechanisms of expression of the three capsid proteins. Mol Plant Pathol.

[6] Vives MC, Martín S, Ambrós S, Renovell A, Navarro L (2008). Development of a full-genome cDNA clone of *Citrus* leaf blotch virus and infection of citrus plants. Mol Plant Pathol.

[7] Wu J, Zhang S, Liang X, Xing F, Atta S (2023). Development and pathogenicity analysis of full-length infectious cDNA clones of citrus yellow mottle-associated virus in *Citrus* plants. J Integr Agric.

[8] Zhao J, Zhang S, Wang J, Lou B, Zhou Y (2024). Construction of an infectious clone of *Citrus* chlorotic dwarf-associated virus and confirmation of its pathogenicity. *Plant Dis*.

[9] Sun Y-D, Folimonova SY (2019). The p33 protein of *Citrus tristeza* virus affects viral pathogenicity by modulating a host immune response. New Phytol.

[10] Yang Z, Zhang Y, Wang G, Wen S, Wang Y (2021). The p23 of *Citrus tristeza* virus interacts with host FKBP-type peptidyl-prolylcis-trans isomerase 17-2 and is involved in the intracellular movement of the viral coat protein. Cells.

[11] Chen AYS, Peng JHC, Polek M, Tian T, Ludman M (2021). Comparative analysis identifies amino acids critical for *Citrus tristeza* virus (T36CA) encoded proteins involved in suppression of RNA silencing and differential systemic infection in two plant species. Mol Plant Pathol.

[12] Ferreira Sa Antunes T, Huguet-Tapia JC, Elena SF, Folimonova SY (2024). Intra-host citrus tristeza virus populations during prolonged infection initiated by a well-defined sequence variant in *Nicotiana benthamiana*. Viruses.

[13] Maccheroni W, Alegria MC, Greggio CC, Piazza JP, Kamla RF (2005). Identification and genomic characterization of a new virus (Tymoviridae family) associated with citrus sudden death disease. J Virol.

[14] Bassanezi RB, Montesino LH, Sanches AL, Spósito MB, Stuchi ES (2007). Effect of *Citrus* sudden death on yield and quality of sweet orange cultivars in Brazil. *Plant Dis*.

[15] Karimi M, De Meyer B, Hilson P (2005). Modular cloning in plant cells. Trends Plant Sci.

[16] Landeo Villanueva S, Malvestiti MC, van Ieperen W, Joosten MHAJ, van Kan JAL (2021). Red light imaging for programmed cell death visualization and quantification in plant-pathogen interactions. Mol Plant Pathol.

[17] Russin WA, Trivett CL (2001). Microwave Techniques and Protocols.

[18] Jambunathan N (2010). Plant Stress Tolerance: Methods and Protocols.

[19] Plumet S, Gerlier D (2005). Optimized SYBR green real-time PCR assay to quantify the absolute copy number of measles virus RNAs using gene specific primers. J Virol Methods.

[20] Pfaffl MW (2001). A new mathematical model for relative quantification in real-time RT-PCR. Nucleic Acids Res.

[21] Mur LAJ, Kenton P, Lloyd AJ, Ougham H, Prats E (2008). The hypersensitive response; the centenary is upon us but how much do we know?. J Exp Bot.

[22] van Wezel R, Dong X, Blake P, Stanley J, Hong Y (2002). Differential roles of geminivirus Rep and AC4 (C4) in the induction of necrosis in *Nicotiana benthamiana*. Mol Plant Pathol.

[23] Nouri S, Zarzyńska-Nowak A, Prakash V (2024). Construction and biological characterization of a cDNA infectious clone of wheat umbra-like virus in wheat and *Nicotiana benthamiana*. Virology.

[24] Bhattacharyya D, Chakraborty S (2018). Chloroplast: the trojan horse in plant-virus interaction. Mol Plant Pathol.

[25] Zhao J, Zhang X, Hong Y, Liu Y (2016). Chloroplast in plant-virus interaction. Front Microbiol.

[26] Dreher TW (2004). Turnip yellow mosaic virus: transfer RNA mimicry, chloroplasts and a C-rich genome. Mol Plant Pathol.

[27] Moriceau L, Jomat L, Bressanelli S, Alcaide-Loridan C, Jupin I (2017). Identification and molecular characterization of the chloroplast targeting domain of turnip yellow mosaic virus replication proteins. Front Plant Sci.

[28] Prod’homme D, Jakubiec A, Tournier V, Drugeon G, Jupin I (2003). Targeting of the turnip yellow mosaic virus 66K replication protein to the chloroplast envelope is mediated by the 140K protein. J Virol.

[29] Torres MA, Jones JDG, Dangl JL (2006). Reactive oxygen species signaling in response to pathogens. Plant Physiol.

[30] Yano A, Suzuki K, Shinshi H (1999). A signaling pathway, independent of the oxidative burst, that leads to hypersensitive cell death in cultured tobacco cells includes a serine protease. Plant J.

[31] Zurbriggen MD, Carrillo N, Hajirezaei MR (2010). ROS signaling in the hypersensitive response: when, where and what for?. Plant Signal Behav.

