# Supplementary material for: Local citrus sudden death-associated virus infection in Nicotiana benthamiana induces chloroplast structural changes and HR-like responses that do not restrict systemic virus movement
Source: J Gen Virol. 2026 Mar 30;107(3):002252. doi: 10.1099/jgv.0.002252 (PMC13035172; doi:10.1099/jgv.0.002252)
Supplement: Uncited Supplementary Material 1. [file jgv-107-02252-s001.pdf]

Local CSDaV infection in *Nicotiana benthamiana* induces chloroplast structural changes and HR-like responses that do not restrict systemic virus movement

Sayuri Iwasaki[1], Dennis van Oevelen[1], Alba Pérez-Sánchez[1], Nell Lakatsch[1], Robin van der Helm[1], Lena Michailidou[1], Nadya N. Icksan[1], Bradley Shibata[2], and Emilyn E. Matsumura[1,*]

Table S1: Primers used in this study. The purpose of each primer is presented.

Primer	Sequence (5'-3')	Purpose
RdRP-CFP_F	GGGGACAAGTTTGTACAAAAAAGCAGGCTTACCCGGGATGGCTTCACCATACTCCAAAGC	Amplification of the RdRP-coding region for in-frame cloning with a C-terminal CFP
RdRP-CFP_R	GGGGACCACTTTGTACAAGAAAGCTGGGTTACCACCAGTGAGAGGCAG	
CFP-RdRP_F	GGGGACAAGTTTGTACAAAAAAGCAGGCTTACCCGGGGCTTCACCATACTCCAAAGC	Amplification of the RdRP-coding region for in-frame cloning with N-terminal CFP
CFP-RdRP_R	GGGGACCACTTTGTACAAGAAAGCTGGGTTTTAACCACCAGTGAGAGGCA	
M13F	CCCAGTCACGACGTTGTAAAACG	Colony PCR
M13R	CACAGGAAACAGCTATGACC	
PRO_F	GGGGACAAGTTTGTACAAAAAAGCAGGCTTACCCGGGTCTGACTGGGATCCTTCCCC	Negative strand cDNA synthesis
CSDaV-RdRP_F	ACGACTCCCTCCTGGATCGT	PCR-based detection of CSDaV
CSDaV-CP_R	ACAGGACCGCCAACAGTGAA	
CSDaV-RdRp_R	GCGTACCGACCCCTTTCTTT	qPCR: CSDaV RNA accumulation
NbPP2a_F	GACCCTGATGTTGATGTTCGCT	qPCR: reference gene
NbPP2a_R	GAGGGATTTGAAGAGAGATTTC	
NbCAT1_F	GTTCTACACCAGAGAGGGAAAC	qPCR: CAT1 expression
NbCAT1_R	GACCATGTCAGGGAACTTCAT	
NbHIN1_F	ATCCTCGGAGTGATTGCATTAG	qPCR: HIN1 expression
NbHIN1_R	TGTTGTTTGTGGTGGACAAATC	
NbPR1_F	TGGTCAATACGGCGAAAAC	qPCR: PR1 expression
NbPR1_R	GAACCCTAGCACATCC	
NbICS1_F	GCAACAGCCAACATAGGTC	qPCR: ICS1 expression
NbICS1_R	TCATAAGAACGGAGGAAACC	

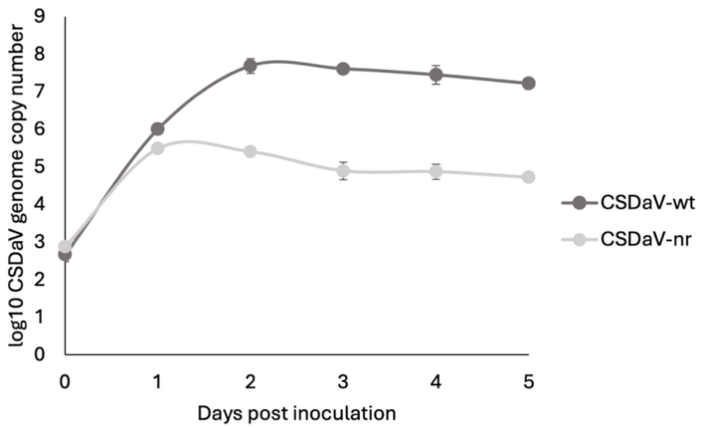

Figure S1: Accumulation of CSDaV genomic copies in *Nicotiana benthamiana* over time following agroinfiltration with wild-type (wt) or non-replicative (nr, negative control) CSDaV clones. Samples were collected at 0, 1, 2, 3, 4, and 5 days post-infiltration (dpi). Reverse transcription quantitative PCR was performed using primers targeting the CSDaV RNA-dependent RNA polymerase (RdRP) gene. The plot shows viral genome copy numbers over time, expressed as $\log_{10}$ values. Data represent the arithmetic mean of five biological replicates.

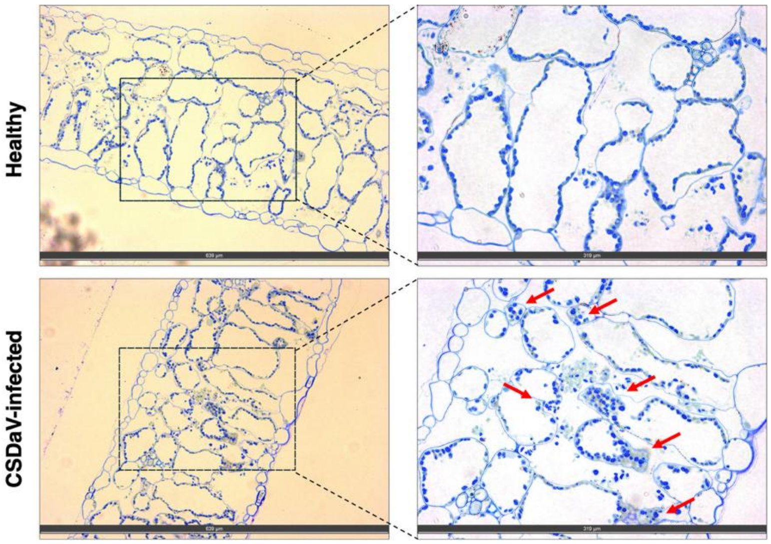

Figure S2: Light microscopy images of toluidine-stained sections from healthy and CSDaV-infected *Nicotiana benthamiana* leaves. Red arrows indicate observed chloroplast clumping.

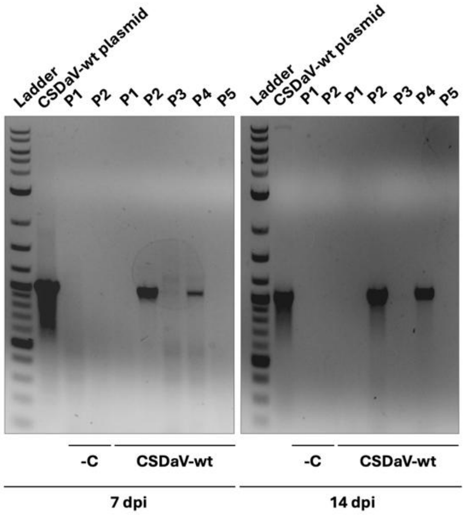

Figure S3: RT-PCR detection of CSDaV positive-sense RNA in upper, non-inoculated leaves of *Nicotiana benthamiana* at 7 and 14 days post-inoculation via *Agrobacterium tumefaciens* carrying CSDaV wild-type (wt) or non-replicative (nr, negative control) clones. P1–P5, individual plant replicates per treatment; –C, plant infiltrated with CSDaV-nr (negative control). Expected PCR product size: 1036 bp.

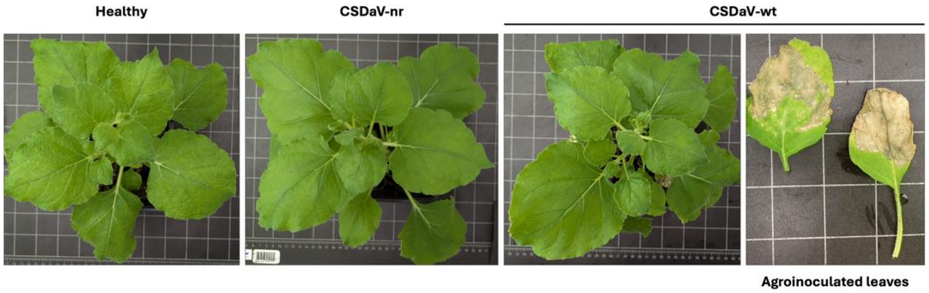

Figure S4: Representative *Nicotiana benthamiana* plants agroinoculated with either the CSDaV wild-type infectious clone (wt) or a non-replicative clone (nr). A non-inoculated (healthy) plant is shown for comparison. Photographs were taken 21 days post-agroinoculation, at which point CSDaV-wt was detected in systemic leaves, although no visible symptoms were observed. Virus-induced necrosis is visible and shown in the agroinoculated leaves of plants infected with CSDaV-wt.

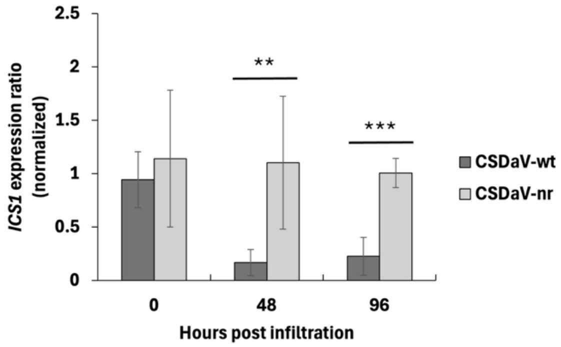

Figure S5: Expression levels of *isochorismate synthase 1* (ICS1), a gene involved with biosynthesis of salicylic acid, in *Nicotiana benthamiana* leaves infiltrated with *Agrobacterium tumefaciens* harboring CSDaV wild-type (wt) or non-replicative (nr, negative control) clones. Gene expression was measured at 0, 48, and 96 hours post infiltration. PP2a was used as reference gene for normalization. Data represent the arithmetic mean of five biological replicates. Data were tested for significant differences ($P \leq 0.05$) using the appropriate unpaired t-test according to data being normally distributed and homogeneity of variances.
